# Anti-caries and Anti-microbial Effects of School-based Fluoride Programs in Myanmar Schoolchildren

**DOI:** 10.3290/j.ohpd.b2960285

**Published:** 2022-04-27

**Authors:** Kaung Myat Thwin, Wa Than Lin, Noboru Kaneko, Kaname Nohno, Hiroshi Ogawa

**Affiliations:** a Assistant Professor, Division of Preventive Dentistry, Faculty of Dentistry and Graduate School of Medical and Dental Sciences, Niigata University, Japan. Study concept and design; collection, analysis and interpretation of the data; wrote the manuscript, gave final approval of the manuscript to be submitted.; b Secretary, Oral Health Education Committee, Myanmar Dental Association (Central), Myanmar. Performed data collection, critically revised and gave final approval of the manuscript to be submitted.; c Senior Lecturer, Division of Preventive Dentistry, Faculty of Dentistry and Graduate School of Medical and Dental Sciences, Niigata University, Japan. Consulted on statistical evaluation, critically revised and gave final approval of the manuscript to be submitted.; d Professor, Division of Oral Science for Health Promotion, Faculty of Dentistry and Graduate School of Medical and Dental Sciences, Niigata University, Japan. Study concept and design, consulted on statistical evaluation, critically revised and gave final approval of the manuscript to be submitted.; e Professor, Division of Preventive Dentistry, Faculty of Dentistry and Graduate School of Medical and Dental Sciences, Niigata University, Japan. Study concept and design, consulted on statistical evaluation, critically revised and gave final approval of the manuscript to be submitted.

**Keywords:** caries experience, caries risk tests, fluoride mouthrinse, fluoride varnish

## Abstract

**Purpose:**

To investigate the anti-caries effects of the school-based fluoride varnish (FV) application at 3-month intervals and weekly fluoride mouthrinsing (FMR) on primary teeth and to evaluate the anti-microbial effects of FV or FMR on cariogenic bacteria among Myanmar children.

**Materials and Methods:**

A 6-month interventional study was conducted on 234 schoolchildren who were divided into three groups: group A received FV application at 3-month intervals, group B received weekly FMR, and group C received no fluoride application. A clinical oral examination and caries risk tests were performed at baseline and the 6-month follow-up.

**Results:**

Caries prevalence and the debris score did not change statistically significantly from baseline to the 6-month follow-up in all groups, whereas the dmfs score statistically significantly increased in group C (p = 0.001). The plaque and saliva scores of Dentocult SM statistically significantly decreased in group A (p = 0.049 and p = 0.006), but those scores statistically significantly increased in group C (p = 0.001 and p = 0.014) after six months. On the other hand, no statistically significant changes were observed in group B. Although the Cariostat scores decreased from baseline to the 6-month follow-up in group A and group B, but increased in group C, no statistically significant differences were observed in any of the groups.

**Conclusion:**

Better anti-microbial effects were obtained for children who received FV application than for those who received FMR, but no statistically significant difference existed between the anti-caries effects of these two approaches.

The prevalence of caries has been declining among children and adolescents in developed countries; however, it still remains an epidemic and highly prevalent condition, especially among underprivileged populations of developing countries.^20,25^ As Myanmar is one of the least developed countries in the world, caries is a serious public health problem in children there.^23^ Based on the results of the first national oral health survey in 2017, only 15.8% of 6-year-old children had no caries in their primary dentition. As early as the age of 6 years, some children already have caries on their newly erupted permanent teeth.^27^ Furthermore, a previous regional survey reported that the prevalence of caries in 5-year-old children was 67.9%, with a mean dmft score of 4.13.^19^ Therefore, caries experience was severe and high among primary schoolchildren in Myanmar. As caries is an increasing burden in Myanmar children, prompt and adequate measures should be applied using evidence-based prevention-oriented methods.

There are various methods of preventing the development of caries in children, such as topical fluoride applications, rinsing with fluoride mouthwash, diet modifications, and oral hygiene care.^7^ Among these methods, topical application of fluoride varnish (FV) is a preferred choice of fluoride for young children and has become the focus of attention because of its effectiveness, safety, and simplicity.^3,11^ It has been widely used in many countries for decades and is particularly advocated for children at moderate or high risk, and for communities in poorly fluoridated areas to prevent and arrest caries.^13^ FV can be applied two to four times a year, and a systematic review reported that topical FV application at 6-month intervals is an effective method of caries prevention among young children, with a caries reduction rate of 33% in primary dentition and 46% in permanent dentition.^26^ Previous reports recommended that FV application at 3-month intervals may provide additional caries preventive benefits for high-risk children.^1,7^ Fluoride mouthrinse (FMR) is also one of the preventive options, and FMR under supervision has been frequently used in school-based programs. The supervised regular use of FMR in young children was proven to be associated with a statistically significant reduction in caries development.^9,14^ A previous study reported that semiannual FV application did not provide superior caries-preventive effects over weekly FMR in school facilities.^8^ However, some studies indicated that a varnish program is associated with a better outcome in terms of reduced caries development compared with a mouthrinse program.^12,21^ More studies are still necessary to confirm their effectiveness in school-based programs. Although studies comparing semi-annual FV application with weekly FMR have been performed, no scientific evidence is available comparing FV application at 3-month intervals with weekly FMR. However, this information is essential to strengthen, facilitate, and concentrate on future public health programs in the prevention of caries.

Caries has a multifactorial aetiology that is triggered by three main factors: host, environment, and bacteria. Mutans streptococci and lactobacilli are well-known cariogenic oral bacteria, in which *Streptococcus mutans* is the most predominant cariogenic species.^22^ The detection and evaluation of cariogenic bacteria is one of the indices for current caries activity and future caries risk.^15^ Therefore, the anti-microbial effects of fluoride on cariogenic bacteria should be considered. A higher amount of fluoride application may contribute to the reduction of dental plaque and suppression of cariogenic bacteria as well as a decrease in acid production.^24^ The long-term use of FMR may reduce the number of mutans streptococci and predominant bacteria in caries development.^28^ However, to date, there are still not many studies on the effect of fluoride on cariogenic bacteria over a given period; thus, no conclusive results are available.

In addition, no research on school-based fluoride programs has been conducted in Myanmar yet. This study aimed to investigate the anti-caries effects of school-based FV application at 3-month intervals and weekly FMR on primary teeth, as well as to evaluate the anti-microbial effects of FV or FMR on cariogenic bacteria among Myanmar children.

## Materials and Methods

### Study Population

The participants of this study were 5-year-old children (1st grade) attending one of the three primary schools in Yangon City, Myanmar. The three primary schools from Yangon city were selected for the sample based on the census sampling method by the Department of Basic Education, Ministry of Education. An invitation letter describing the purpose and procedures of the study was sent to parents or caregivers of the children through the principals of primary schools. Parents or caregivers who agreed to participate in the study signed and returned a written consent form.

A total of 295 schoolchildren agreed to participate in the study. Children who had no history of fluoride application, who also obtained parental consent to participate, and were in good health were eligible for this study. Among the 270 children who met the inclusion criteria, five were excluded because they had a medical history of systemic diseases, long-term medications, and uncooperative behaviour. The study started with 265 children, and 31 children dropped out during the 6-month follow-up because they were absent on the days of examination or moved to other schools. Therefore, the final number of children who completed the study from October 2019 to April 2020 was 234.

This study was approved by the Department of Basic Education, Ministry of Education, Myanmar (Reg. No. 491/Health Survey/19/2019). The study protocol was also approved by the Ethics Committee of Niigata University, Japan (Approval No. 2019-0256).

### Study Design

**Fig 1:**
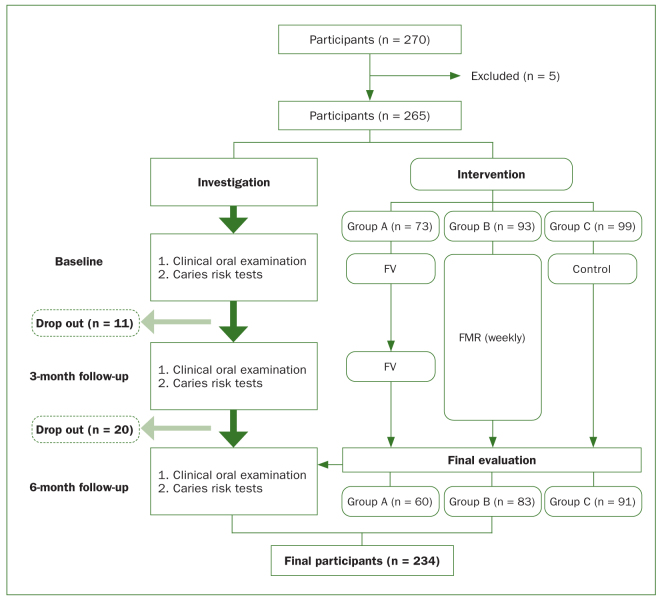
Flowchart of the study protocol.

Figure 1 shows the flowchart of this 6-month interventional study. In the process of group assignment, the children of three primary schools were allocated to three groups by matching their clinical and microbial oral health status at the school level: one school for each group. Participants in group A received FV at 3-month intervals, those in group B received weekly FMR, and group C was the control group. In order to minimise any examiner bias, a collaborative research team with their specific roles was formed, and the clinical oral examinations and microbial evaluations were performed by blinded examiners.

### Clinical Oral Examination

All participating children were clinically examined for oral health status at baseline and during the 6-month follow-up. A dentist conducted all the clinical oral examinations, and an assistant helped record the data for their respective schools. Calibration of examination criteria and intervention procedures were done prior to study inception.

Dentition and oral hygiene status were assessed using a dental mirror, a WHO-CPI probe, and a handheld light, while sitting knee-to-knee. Four surfaces (labial, lingual, mesial, and distal) of anterior teeth and five surfaces (buccal, lingual, mesial, distal, and occlusal) of posterior teeth were assessed based on the WHO criteria for caries. Oral hygiene status was evaluated on six index teeth (the buccal/labial surfaces of teeth 55 or 54, 51, 65 or 64, and 71; and the lingual surfaces of teeth 75 or 74 and 85 or 84) based on the criteria of the simplified oral hygiene index (OHI-S).^6^ The scores for each tooth surface were summed and divided by the number of teeth examined to calculate the debris score. The debris index scores were trichotomized: 1: debris score < 1; 2: 1 ≤ debris score < 2; 3: debris score ≥ 2.

### Caries Risk Tests

After oral examination, caries risk tests were performed by using Dentocult SM (Oral Care; Tokyo, Japan) and Cariostat (Dentsply-Sankin; Tokyo, Japan) at baseline and the 6-month follow-up. The Dentocult SM test is a popular method for determining the bacterial count (colony forming units, CFU) of mutans streptococci in dental plaque and saliva.^24^ The Cariostat method, a colorimetric caries risk-assessment test based on color changes, is also a reliable and good predictor for measuring caries activity by detecting the degree of acid production by cariogenic bacteria in the dental plaque.^18^ The plaque sample for Dentocult SM was obtained from four tooth surfaces (buccal surfaces of teeth 55 or 54 and 51, and lingual surfaces of teeth 71 and 74 or 75) using a toothpick, and from saliva by pressing a strip on the dorsum of the tongue five times. For Cariostat, the plaque sample was collected from buccal surfaces of teeth 54 and 55 by wiping four to five times with a sterile cotton swab. With support from a microbiologist, the caries risk-test vials were incubated at 37°C for 48 h, and scores ranging from 0 to 3 were evaluated according to the manufacturer’s instructions. The Dentocult SM plaque score was calculated by adding the scores of four tooth surfaces and dividing by four.

The children were divided into two groups (no change/increased score; decreased score) based on the changes in caries-risk test scores between baseline and the 6-month follow-up appointment.

### FV and FMR Application

Prior to fluoride applications, a toothbrush was given to each child, and they were asked to brush their teeth only with water. FV (5%, 22,600 ppmF) (Denu clear varnish, HDI Dental; Seoul, South Korea) and FMR (0.2%, 900 ppmF) (Miranol, Bee Brand Medico Dental; Osaka, Japan) were used for the intervention in this study. In group A, the tooth surfaces were dry and isolated with the cotton rolls. A thin layer of FV (2.5 ml) was applied onto all erupted sound tooth surfaces with an applicator and maintained for 30 s. In group B, a dentist taught the schoolteachers and children the correct method of mouthrinsing prior to FMR procedures; the children in group B practiced rinsing with drinking water first for a month. After practicing initial mouthrinsing with drinking water, children started FMR solution using 10 ml of 0.2% NaF solution under the supervision of schoolteachers. The rinsing solution used in this study was prepared by dissolving 1 package of FMR (1.8 g) with 100 ml of water in a plastic container to make a solution with a concentration of 0.2% NaF. Ten milliliters of the prepared solution was measured accurately into each paper cup at the school. A paper cup containing the prepared solution was given to each child, and then the children were asked to rinse vigorously for 1 min. The children from both groups avoided eating and drinking for one hour.

### Statistical Analysis

Data were analysed using SPSS 22.0 (SPSS; Chicago, IL, USA). Descriptive statistics were computed to report the means or proportions of oral health status and caries risk tests. Statistical tests such as the chi-squared test, McNemar’s test, or ANOVA were used for data analysis. The threshold for statistical significance for all tests was set at p < 0.05.

## Results

### Baseline Characteristics of the Three Groups

At the baseline, there were no statistically significant differences in mean age, proportions of boys and girls, or numbers of teeth present between the three groups. No statistically significant differences were found in caries prevalence, mean numbers of decayed (ds), missing (ms), and filled tooth surfaces (fs) (dmfs), debris score, and scores for Dentocult SM and Cariostat tests between the three groups at baseline (Table 1). The caries-risk test scores were significantly associated with caries prevalence (p < 0.001) and mean dmfs in all groups (p < 0.001).

**Table 1:** Characteristics of the three groups at baseline

	Group A, FV	Group B, FMR	Group C, Control	p-value
	(n = 60)	(n = 83)	(n = 91)	
Caries prevalence	48 (80.0%)	67 (80.7%)	79 (87.1%)	0.302
ds	12.50 ± 15.94	11.05 ± 10.55	11.66 ± 11.44	0.456
ms	0.13 ± 0.79	0.49 ± 3.16	0.21 ± 1.08	0.439
fs	0.20 ± 1.11	0.06 ± 0.33	0.21 ± 0.73	0.377
dmfs	12.83 ± 16.01	11.60 ± 11.85	12.53 ± 11.54	0.744
Debris score	1.46 ± 0.37	1.39 ± 0.42	1.52 ± 0.36	0.745
Dentocult SM (plaque)	0.92 ± 0.43	0.88 ± 0.38	0.95 ± 0.45	0.152
Dentocult SM (saliva)	1.34 ± 0.94	1.17 ± 0.81	1.29 ± 0.80	0.255
Cariostat	1.26 ± 0.54	1.17 ± 0.58	1.25 ± 0.55	0.390

ds: decayed primary tooth surfaces, ms: missing primary tooth surfaces, fs: filled primary tooth surfaces, dmfs: decayed, missing, filled primary tooth surfaces. Caries prevalence: number (%); chi-squared test. Other variables: mean ± standard deviation, ANOVA.

### Baseline vs 6-month Follow-up

Table 2 shows the comparison of clinical oral health status and caries-risk test scores between baseline and the 6-month follow-up in the three groups. The prevalence of caries slightly increased from baseline to the 6-month follow-up appointment in all groups; however, the difference was not statistically significant. The mean dmfs scores did not differ statistically significantly in groups A and B at the 6-month follow-up appointment compared with baseline. On the other hand, the mean dmfs score statistically significantly increased from baseline to the 6-month follow-up appointment in group C (p = 0.001). The debris scores decreased from 1.46 ± 0.37 at baseline to 1.41 ± 0.39 at the 6-month follow-up in group A (p = 0.419) and from 1.39 ± 0.42 to 1.36 ± 0.33 in group B (p = 0.145); however, it increased from 1.52 ± 0.36 at baseline to 1.66 ± 0.38 at the 6-month follow-up in group C (p = 0.604). There were no statistically significant differences in debris score between baseline and the 6-month follow-up in all groups.

**Table 2:** Comparison between baseline and the 6-month follow-up appointment

	Group A, FV (n = 60)			Group B, FMR (n = 83)			Group C, Control (n = 91)		
	Baseline	6-month	p-value	Baseline	6-month	p-value	Baseline	6-month	p-value
Caries prevalence	80.0%	84.0%	0.795	80.7%	84.0%	0.701	87.1%	95.1%	0.180
dmfs	12.83 ± 16.01	14.82 ± 17.79	0.061	11.60 ± 11.85	14.47 ± 12.66	0.052	12.53 ± 11.54	17.08 ± 11.51	0.001
Debris score	1.46 ± 0.37	1.41 ± 0.39	0.419	1.39 ± 0.42	1.36 ± 0.33	0.145	1.52 ± 0.36	1.66 ± 0.38	0.604
Dentocult SM (plaque)	0.92 ± 0.43	0.80 ± 0.27	0.049	0.86 ± 0.38	0.76 ± 0.34	0.178	0.95 ± 0.45	1.20 ± 0.39	0.001
Dentocult SM (saliva)	1.34 ± 0.94	0.96 ± 0.88	0.006	1.17 ± 0.81	0.99 ± 0.87	0.142	1.29 ± 0.80	1.48 ± 0.84	0.014
Cariostat	1.26 ± 0.54	1.24 ± 0.65	0.542	1.17 ± 0.58	1.05 ± 0.65	0.645	1.25 ± 0.55	1.40 ± 0.55	0.124

Caries prevalence: %; McNemar’s test. Other variables: mean ± standard deviation; repeated measures ANOVA.

In group A, the plaque and saliva scores of Dentocult SM statistically significantly decreased from baseline to the 6-month follow-up appointment (p = 0.049 and p = 0.006). The Cariostat score also decreased from baseline to the 6-month follow-up appointment, although no statistically significant difference was found (p = 0.542). In group B, all caries risk scores decreased from baseline to the 6-month follow-up, but no statistically significant differences were observed. On the other hand, the plaque and saliva scores of Dentocult SM statistically significantly increased during the 6 months of follow-up in group C (p = 0.001 and p = 0.014). The Cariostat score also increased from baseline to the 6-month follow-up appointment, although the difference was not statistically significant (p = 0.124).

### Comparison of Changes between the Three Groups

The mean or distributional changes between the three groups are shown in Table 3. Less than half of the children in groups A and B (46.7% and 47.0%, respectively) developed carious lesions during the 6-month follow-up period, while approximately two-thirds of the children (67.0%) in group C developed carious lesions. The development of caries was statistically significantly greater in group C than in groups A and B at the 6-month follow-up (p = 0.010). In addition, children in groups A and B had statistically significantly improved plaque and saliva scores of Dentocult SM compared to those in group C (p < 0.001 and p < 0.001). On the other hand, the changes in mean dmfs score, debris score, and Cariostat score did not differ statistically significantly between the three groups.

**Table 3:** Comparison of changes between the three groups

	Group A, FV	Group B, FMR	Group C, Control	p-value
	(n = 60)	(n = 83)	(n = 91)	
Caries development	28 (46.7%)*	39 (47.0%)*	61 (67.0%)	0.010
⊿dmfs	2.00 ± 16.94	2.87 ± 11.79	4.55 ± 11.52	0.090
Children who improved in				
Debris score	29 (48.3%)	43 (51.8%)	32 (35.2%)	0.179
Dentocult SM (plaque)	22 (36.7%)*	37 (44.6%)*	15 (16.5%)	< 0.001
Dentocult SM (saliva)	31 (51.7%)*	34 (41.0%)*	16 (17.6%)	< 0.001
Cariostat	20 (33.3%)	24 (28.9%)	16 (17.6%)	0.066

⊿dmfs = dmfs (6-month) – dmfs (baseline); ⊿dmfs: mean ± standard deviation; ANOVA. Other variables: number (%); chi-squared test. *Statistically significantly different from Group C (chi-squared test with Bonferroni correction).

### Comparison of Caries Status Based on Changes in Caries Risk Scores

Table 4 shows the mean or distributional changes of dental caries based on the two tests in the three groups. In children with a decreased score according to Dentocult SM (plaque), caries development was statistically significantly lower in group A than in groups B and C (p = 0.048). For the saliva scores of Dentocult SM, caries development and the mean dmfs score changes did not differ statistically significantly between the groups A, B and C in both ‘no change/increased score’ and ‘decreased score’ groups. On the other hand, caries development in group C statistically significantly worsened from baseline to the 6-month follow-up in children with an increased score according to Cariostat (p = 0.042).

**Table 4:** Comparison of caries status based on changes in caries risk tests

			Group A, FV	Group B, FMR	Group C, Control	p-value
			(n = 60)	(n = 83)	(n = 91)	
Dentocult SM (plaque)						
	No change / increased score		(n = 38)	(n = 46)	(n = 76)	
		Caries development	22 (57.9%)	24 (52.2%)	52 (68.4%)	0.181
		⊿dmfs	3.86 ± 14.56	6.26 ± 12.17	5.58 ± 12.26	0.287
	Decreased score		(n = 22)	(n = 37)	(n = 15)	
		Caries development	6 (27.3%)	15 (40.5%)	9 (60.0%)	0.048
		⊿dmfs	1.73 ± 13.88	5.38 ± 11.99	4.55 ± 12.96	0.393
Dentocult SM (saliva)						
	No change / increased score		(n = 29)	(n = 49)	(n = 75)	
		Caries development	19 (65.5%)	27 (55.1%)	52 (69.3%)	0.267
		⊿dmfs	3.84 ± 15.82	5.83 ± 12.29	7.49 ± 11.37	0.101
	Decreased score		(n = 31)	(n = 34)	(n = 16)	
		Caries development	9 (29.0%)	12 (35.3%)	9 (56.3%)	0.180
		⊿dmfs	1.00 ± 12.58	3.53 ± 10.86	4.61 ± 12.72	0.472
Cariostat						
	No change / increased score		(n = 40)	(n = 59)	(n = 75)	
		Caries development	19 (47.5%)	30 (49.2%)	52 (69.3%)	0.042
		⊿dmfs	4.03 ± 10.95	6.86 ± 10.98	5.23 ± 10.53	0.198
	Decreased score		(n = 20)	(n = 24)	(n = 16)	
		Caries development	7 (35.0%)	10 (41.7%)	9 (56.3%)	0.432
		⊿dmfs	0.13 ± 8.70	3.42 ± 12.50	5.15 ± 12.56	0.354

Caries development: number (%); chi-squared test. ⊿dmfs: mean ± standard deviation; ANOVA.

## Discussion

To the best of our knowledge, this is the first interventional study to evaluate the anti-caries and anti-microbial effects of FV application at 3-month intervals vs weekly FMR in Myanmar children. Inadequate exposure to fluoride has been reported to be one of the major reasons for the burden of caries in most countries.^17^ Fluoride works to prevent caries primarily through topical mechanisms, such as the inhibition of demineralisation, enhancement of remineralisation, and inhibition of bacterial metabolism.^5^ For countries with limited oral health resources and high caries prevalence, such as Myanmar, establishing an effective school-based fluoride program is financially and materially critical for tackling the problem of high caries experience in children.

The overall caries prevalence in the study was approximately 83%. Caries prevalence apparently increased from baseline to 6-month follow-up in group C, although it did not differ statistically significantly between children with and without fluoride applications. The dmfs score of children who received fluoride applications of any kind showed no statistically significant changes from baseline to the 6-month follow-up; however, those in the control group showed statistically significantly higher dmfs. The findings of this study are in line with those of previous studies, which demonstrate the effectiveness of fluoride in caries prevention.^1,24,26,28^ Children without any fluoride exposure might develop caries within six months. Fluoride has been found to be the most effective cariostatic agent in dentistry. Fluoride becomes incorporated into the tooth structure and makes it more resistant to dissolution by acids. The ionic fluoride in saliva, dental plaque, and within enamel and dentin shifts the demineralisation-remineralisation equilibrium toward remineralisation.^5^ Therefore, the administration of fluoride is a unique opportunity for caries prevention; thus, adequate and effective preventive measures, including the use of fluoride, should be promptly implemented in Myanmar schoolchildren. The debris score showed the clinical differences between children with or without any fluoride applications, although these were not statistically significant. It decreased from baseline to the 6-month follow-up in children who received FV or FMR, but increased in participants in the control group. This was probably because cationic fluoride preparations could reduce the acidogenic capacity of dental plaque formation.^24^

Although many studies have investigated the effectiveness of fluoride on hard dental tissues, little attention has been paid to its effects on cariogenic bacteria. In this study, the effects of fluoride on *Streptococcus mutans* count in dental plaque and saliva, as well as the degree of acid production by cariogenic bacteria, were analysed. The overall scores of all caries risk tests decreased from baseline to the 6-month follow-up in children who received FV or FMR, but increased in those who did not receive any fluoride applications. It is widely accepted that fluoride acts in multiple ways to affect the metabolism of cariogenic bacteria.^16^ The amount of mutans streptococci in dental plaque and saliva significantly decreased after 6 months in children with FV application, whereas no significant change was observed in children with FMR application. The results showed that FV application at 3-month intervals had a superior antibacterial effect on the mutans streptococci count compared with weekly FMR. *Streptococcus mutans* is a microorganism unusually sensitive to fluoride, because of the direct inhibitory effect of fluoride on its proton-transporting ATP-ase system.^16^ Higher amounts of fluoride from FV can inhibit the plaque acid production by means of a direct inhibitory effect on the metabolic activity of cariogenic bacteria and the reduction of other species in the oral cavity.^2,18^

The mean or distributional changes of caries status, debris score, and level of cariogenic bacteria were evaluated in this study. The proportion of children who developed carious lesions was statistically significantly lower in children who received FV or FMR than in those who did not receive any fluoride applications. Moreover, children in groups A and B had statistically significantly improved plaque and saliva scores of Dentocult SM compared to those in group C. Cariogenic bacteria play a crucial role in caries development. The colonisation of teeth by cariogenic bacteria was found to be one of the most important associated risk factors for caries, with *Streptococcus mutans* being the primary species associated with the early cariogenic process. The main finding of our study was that the antibacterial effect of fluoride may be significant both in preventing initial carious lesions and in enhancing the remineralisation capacity.^16^

Comparison of caries status based on changes in caries risk scores proved that more favourable findings were obtained for children who received FV than for those who received FMR or no fluoride application. The improvements in children with FV application were demonstrated to change the cariogenic potential. At low concentrations, fluoride is bacteriostatic, and at high concentrations, it is bactericidal.^4^ FV used in this study contains 5% sodium fluoride (22,600 ppmF); after application, fluoride is slowly released to the oral biofilm over a certain period.^2^ Therefore, a higher concentration of fluoride and the constant presence of fluoride ions in the oral cavity were found to hamper bacterial acid production, decrease the rate of demineralisation, modify the physicochemical properties of teeth, and decrease plaque accumulation to a greater extent than did a lower concentration of fluoride.^10,24^

The present study had some limitations. For instance, the study sample could not represent the whole population of 5-year-old Myanmar schoolchildren, because it was conducted only in three primary schools of Yangon city. We also did not investigate whether the participants of this study used fluoridated toothpaste; that could possibly influence the actions of FV or FMR applications. In addition, because of the coronavirus pandemic, this study could be performed only for a duration of 6 months, although it was meant to last for 1 year. However, this 6-month interventional study provides useful information about the comparative effects of the caries prevention between FV application at 3-month intervals and weekly FMR and their anti-bacterial effects on cariogenic bacteria.

## Conclusion

Fluoride application is most effective in preventing caries. The application of FV over a 3-month interval was observed to have greater anti-microbial effects than weekly FMR, although there were no statistically significant differences in their caries preventive effects. The data presented here suggested that frequent FV application should be utilised more for high-risk children. However, because of its relatively high material costs per application, its cost effectiveness might hamper its widespread adoption by public oral health care services, especially in low-income countries. Further studies thus would be necessary to provide scientific evidence for cost-effectiveness of school-based fluoride programs.

Furthermore, this study revealed that caries experience and oral hygiene status were influenced by cariogenic bacteria. Therefore, it is important to reduce the number of cariogenic bacteria and the degree of acid production to prevent caries.
